# The effects of surface spin on magnetic properties of weak magnetic ZnLa_0.02_Fe_1.98_O_4_ nanoparticles

**DOI:** 10.1186/1556-276X-9-545

**Published:** 2014-10-02

**Authors:** Shitao Xu, Yongqing Ma, Yuanfeng Xu, Xiao Sun, Bingqian Geng, Ganhong Zheng, Zhenxiang Dai

**Affiliations:** 1 Anhui Key Laboratory of Information Materials and Devices, School of Physics and Materials Science, Anhui University, Hefei 230039, People’s Republic of China; 2 School of Physics and Electronic Information, Huaibei Normal University, Huaibei 235000, People’s Republic of China

**Keywords:** Nanoparticles, Ferrite, Surface spin, Magnetic properties

## Abstract

In order to prominently investigate the effects of the surface spin on the magnetic properties, the weak magnetic ZnLa_0.02_Fe_1.98_O_4_ nanoparticles were chosen as studying objects which benefit to reduce as possibly the effects of interparticle dipolar interaction and crystalline anisotropy energies. By annealing the undiluted and diluted ZnLa_0.02_Fe_1.98_O_4_ nanoparticles at different temperatures, we observed the rich variations of magnetic ordering states (superparamagnetism, weak ferromagnetism, and paramagnetism). The magnetic properties can be well understood by considering the effects of the surface spin of the magnetic nanoparticles. Our results indicate that in the nano-sized magnets with weak magnetism, the surface spin plays a crucial rule in the magnetic properties.

## Background

Ferrite nanocrystals have been extensively studied due to their tunable and remarkable magnetic properties as well as catalytic properties not existing in the corresponding bulk materials [1-5]. In the fundamental research field, magnetic nanoparticles (NPs) usually serve as ideal model systems, e.g., the Stoner-Wohlfarth [6,7] and Néel-Brown model [8], or to study the finite-size effect [9]. As the size of a magnetic particle decreases, the significance of the surface spins increases, resulting in the various magnetic ordering states such as spin-glass or cluster-glass-like behavior [10-13] or weak ferromagnetism [14,15] of the surface spins.

Compared with strong magnetic materials which have higher *H*_*c*_ and *M* values, the material with weak magnetism (small values of coercivity *H*_*c*_ and magnetization *M*) is a good candidate for studying the effects of surface spins on magnetic properties, because the strong anisotropy or interparticle dipolar interaction in the strong magnetic system can suppress the effects of surface spin [16,17]. ZnFe_2_O_4_ crystallizes in the bulk in the *normal* spinel structure with Fe^3+^ ions (5 *μ*_*B*_ moment per Fe^3+^ ion) occupying octahedral sites and Zn^2+^ ions (with zero moment) occupying tetrahedral sites. Superexchange interaction between two Fe^3+^ ions will have their moments aligned anti-parallel to each other which results in the antiferromagnetic (AFM) ZnFe_2_O_4_ [18,19] with the theoretical moment being 0 *μ*_*B*_*/f.u*. For the La^3+^ substituted bulk ZnLa_0.02_Fe_1.98_O_4_ (ZLFO), the theoretical moment is 0.1 *μ*_*B*_*/f.u.*, which exhibits the weak ferromagnetism. In the present work, we take ZLFO as a studying object to investigate the effects of surface spins on the magnetic properties. We first prepared ZLFO NPs via a hydrothermal method. Then, some of the NPs were diluted in the Al_2_O_3_ matrix and others were undiluted, both followed by annealing at different temperatures of 700°C, 800°C, 900°C, and 1,000°C. The magnetic measurements show that the ZLFO NPs have the weak magnetism with small *M* and *H*_*c*_ values. Our results indicate that the surface spins significantly affect the macromagnetism of ZLFO NPs.

### Experimental details

#### Material preparation

All raw materials include: iron (III) nitrate hexahydrate [Fe(NO_3_)_3_ · 6H_2_O, 99%], zinc nitrate hexahydrate [Zn(NO_3_)_2_ · 6H_2_O, 99%], lanthanum (III) acetate sesquihydrate [La(OOCCH_3_)_3_ · 1.5H_2_O, 99.9%], and aluminum nitrate nonahydrate [Al(NO_3_)_3_ · 9H_2_O], serving as the sources of metallic ions in ZLFO and Al_2_O_3_; sodium acetate trihydrate (C_2_H_3_NaO_2_ · 3H_2_O, 99%) and 1-hexadecyltrimethylammonium bromide (C_19_H_42_BrN, 99%), being used as surfactants for improving precursor’s dispersibility; ethylene glycol (C_2_H_6_O_2_, 99%), acting as the solvent.

Firstly, 36 mmol Zn(NO_3_)_2_ · 6H_2_O, 72 mmol Fe(NO_3_)_3_ · 6H_2_O, 7.2 mmol La(OOCCH_3_)_3_ · 1.5H_2_O, and 108 mmol C_2_H_3_NaO_2_ · 3H_2_O were dissolved in 300-mL anhydrous C_2_H_6_O_2_ with magnetic stirring. Then, 1.08 mmol C_19_H_42_BrN was added into the solution with continuous stirring at 40°C for 30 min to get a homogeneous solution. Subsequently, the solution was transferred into 50-ml Teflon-lined stainless steel autoclave and maintained at 200°C for 24 h to obtain the ZLFO NPs. The typical synthesis procedure can be shown by the following [20]:

Zn(NO_3_)_2_ · 6H_2_O + 0.02 La(OOCCH_3_)_3_ · 1.5H_2_O + 1.98 Fe(NO_3_)_3_ · 6H_2_O + C_2_H_3_NaO_2_ · 3H_2_O + C_2_H_6_O_2_ → ZnLa_0.02_Fe_1.98_(OOCH_2_CH_3_)_8_ · nH_2_O + NaNO_3_.

ZnLa_0.02_Fe_1.98_(OOCH_2_CH_3_)_8_ · nH_2_O will decompose above 200°C and produce ZLFO.

After the autoclave was cooled down to room temperature naturally, the precipitate obtained was separated by centrifugation, washed with distilled water and anhydrous ethanol several times, and subsequently dried at 80°C. The obtained ZLFO NPs were divided into two parts. One is diluted in the Al_2_O_3_ matrix and the other is undiluted.

ZLFO NPs were added to the solution of Al(NO_3_)_3_ · 9H_2_O and ethanol under sonicating with mass ratio of ZLFO:Al_2_O_3,_ being 3:2. Then, the mixture was dried at 80°C. The undiluted and diluted ZLFO were both divided equally into four parts for annealing at 700°C, 800°C, 900°C, and 1,000°C for 2 h to obtain the final samples, which are hereafter referred to as UD700, UD800, UD900, and UD1000 for undiluted samples and D700, D800, D900, and D1000 for diluted samples, respectively.

The crystal structure was characterized by X-ray diffraction analysis using an X-ray diffractometer (XRD; DX-2000 SSC) with Cu Kα irradiation (λ = 1.5418 Å) from 10° to 80° with a step of 0.02°. The magnetic measurements were carried out by Quantum Design superconducting quantum interference device (SQUID) MPMS system(PPMS EC-II) (Quantum Design, San Diego, CA, USA). High-resolution transmission electron microscopy (HRTEM) (JEOL JEM-2100, JEOL, Akishima-shi, Tokyo, Japan) was used to observe the morphology, selected area electronic diffraction (SAED), and lattice fringes.

## Results and discussion

### Structural characterization

**Figure 1:**
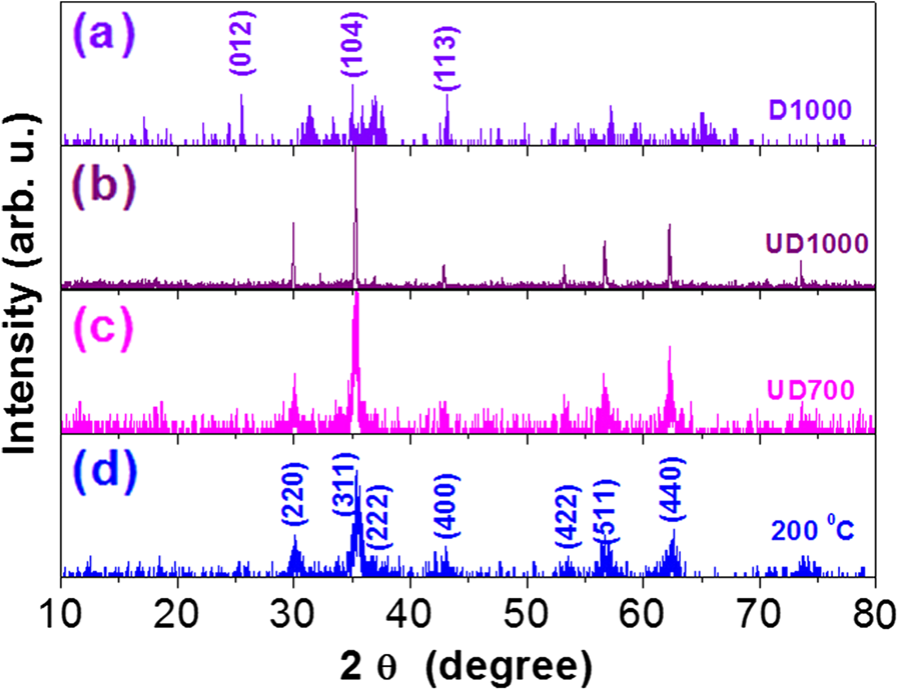
**XRD patterns.** XRD patterns for the as-prepared sample at 200°C **(d)**, samples UD700 **(c)**, UD1000 **(b)**, and D1000 **(a)**, respectively.

Firstly, the crystal structures of the obtained samples are analyzed. Figure 1 representatively shows XRD patterns for the as-prepared sample at 200°C (d), UD700 (c), UD1000 (b), and D1000 (a). According to the standard PDF card (No.79-1150), the diffraction peaks of the as-prepared sample can be indexed to the cubic spinel Zn ferrite with space group *Fd3m* (227) as reported in literature [21]. For the undiluted samples UD700 and UD1000, the intensity of diffraction peak increases with increasing the annealing temperature. The cell lattice parameter *a* of ZLFO can be obtained from $\sin^{2}\theta =\frac{\lambda^{2}}{4a^{2}}\left(H^{2}+K^{2}+L^{2}\right)$, where *a* is the lattice parameter, *θ* the diffraction angle, *λ* the wavelength of the Cu *Kα* irradiation, and (*HKL*) the crystal plane index. The obtained lattice parameter *a* is 0.8339, 0.8424, and 0.8437 nm for the as-prepared, UD700 and UD1000, respectively. Correspondingly, the X-ray density *d*_*x*_ can be calculated by the formula *d*_*x*_ = 8 *M*/*Na*^3^, where *M*, *N*, and *a* are the molecular weight, Avogadro’s number, and lattice parameter, respectively [21]. The calculated *d*_*x*_ value is 5.56, 5.39, and 5.37 g/cm^3^, comparable to 5.30 g/cm^3^ [22] and 5.35 g/cm^3^ for ZnFe_2_O_4_ [23]. The average crystallite grain size, calculated from the (311) XRD peak using Debye Scherrer’s formula, is 9.5, 10.7, and 38.8 nm.

For the diluted sample D1000, as shown in Figure 1a, the diffraction peaks of ZLFO cannot be observed, indicating that ZLFO was deeply embedded in the Al_2_O_3_ matrix. Several diffraction peaks of (012), (104), and (113) facets can be attributed to the reflection of Al_2_O_3_.

**Figure 2:**
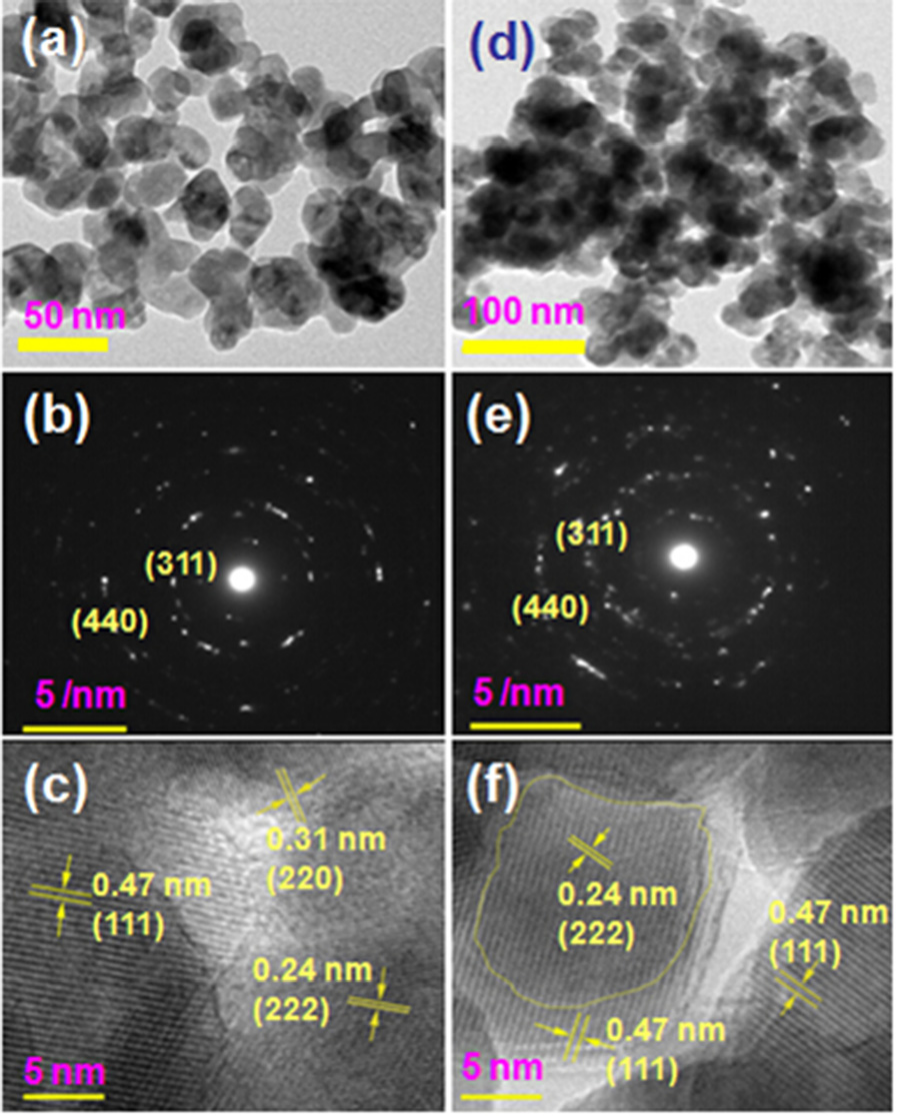
**TEM, SAED, and HRTEM images of the samples.** TEM **(a and d)**, SAED **(b and e)**, and HRTEM **(c and f)** images of the samples UD700 (*left panel*) and D700 (*right panel*).

Figure 2 shows the TEM (a and d), SAED (b and e), and HRTEM (c and f) images of the samples UD700 (left panel) and D700 (right panel). The sample UD700 consists of particles with the size about 50 nm and some particles agglomerate to larger particles, as shown in Figure 2a. The SAED image in Figure 2b exhibits the distinct diffraction circles indicating the characteristic of polycrystalline. As seen in Figure 2c, the interfringe distances of 0.24, 0.31, and 0.47 nm correspond to the (222), (220), and (111) crystalline planes of ZLFO. The particles in the diluted sample D700 are conglomerated, as shown in Figure 2d, and the SAED image in Figure 2e appears some random diffraction dots from Al_2_O_3_. The HRTEM image exhibits distinct fringes with distances being 0.24 and 0.47 nm, also corresponding to (222) and (111) crystalline planes of ZLFO.

### Magnetic properties

**Figure 3:**
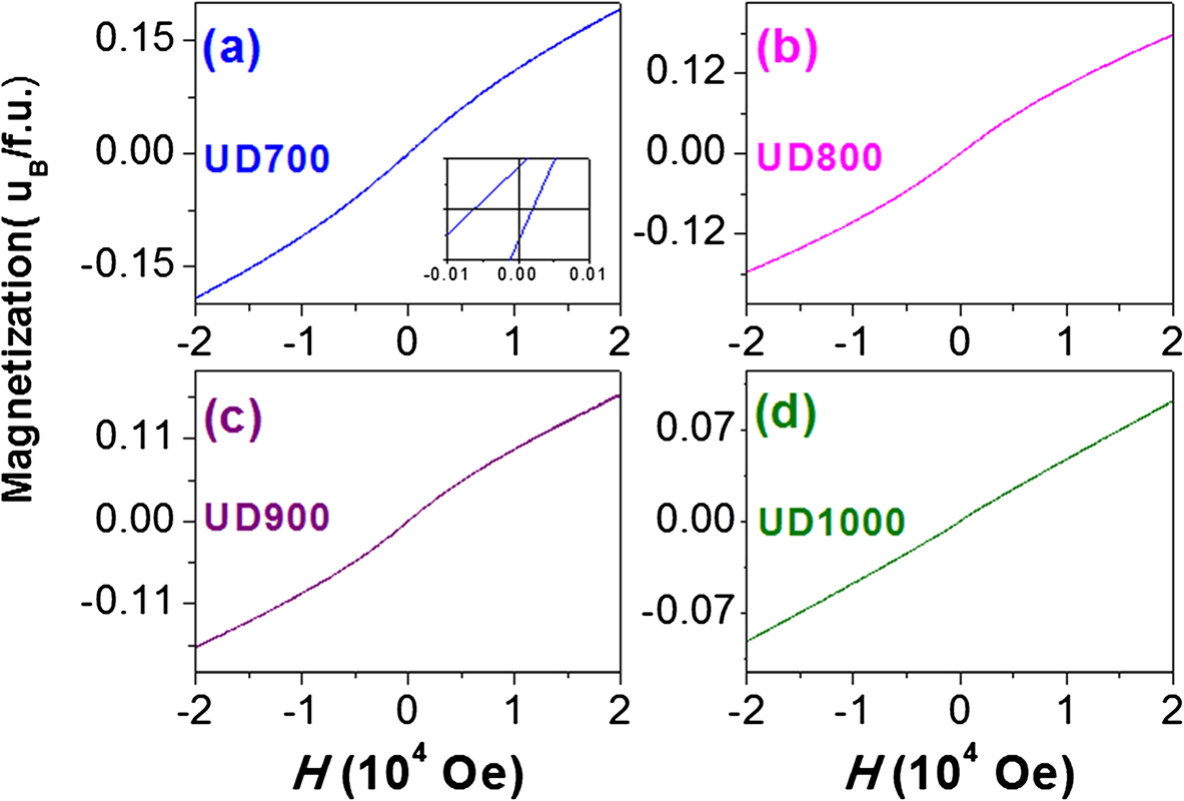
**The dependence of magnetization on magnetic field.** The magnetization (*M*) dependence on the applied magnetic field (*H*) (-2 T < *H* < +2 T), viz*M*(*H*) loops of all undiluted samples UD700 **(a)**, UD800 **(b)**, UD900 **(c)**, and UD1000 **(d)** measured at room temperature. The inset of **(a)** shows the magnified plot of loop in the low *H* region.

Figure 3 shows the magnetization (*M*) dependence on the applied magnetic field (*H*) (-2 T < H < +2 T), viz *M*(*H*) loops of all undiluted samples, measured at room temperature. The loop shape of the samples UD700, UD800, and UD900 resembles to that observed in ZnFe_2_O_4_ [24], being characteristic of the superparamagnetism (SPM) with very small coercivity *H*_*c*_ (<80 Oe). The *H*_*c*_ value is 62, 68, 80, and 52 Oe for UD700, UD800, UD900, and UD1000, respectively. As shown in the inset of Figure 3a, a distinct loop shift along *H* axis, i.e., the exchange-bias effect, can be observed, implying the existence of the surface spin layer (SSL) of the NP [25-28]. The *M*(*H*) loop of the sample UD1000 is almost a straight line, behaving as the paramagnetism (PM). The maximum magnetization (*M*_max_) at *H* = 2 T is 0.19, 0.18, 0.17, and 0.09 *μ*_*B*_*/f.u.* for UD700, UD800, UD900, and UD1000, respectively. The sample UD700 has the highest moment of 0.19 *μ*_*B*_*/f.u.*, larger than the theoretical value of 0.1 *μ*_*B*_*/f.u.* for the 2% La^3+^ substituted bulk ZLFO. Such a larger *M*_max_ value can also be assigned to the contribution of SSL [29]*.* With increasing the annealing temperature, the *M*_max_ monotonously decreases. Therefore, the total moment of NPs are contributed by the moments of particle core (*M*_core_) and SSL (*M*_SSL_), i.e., the core-shell magnetization model.

**Figure 4:**
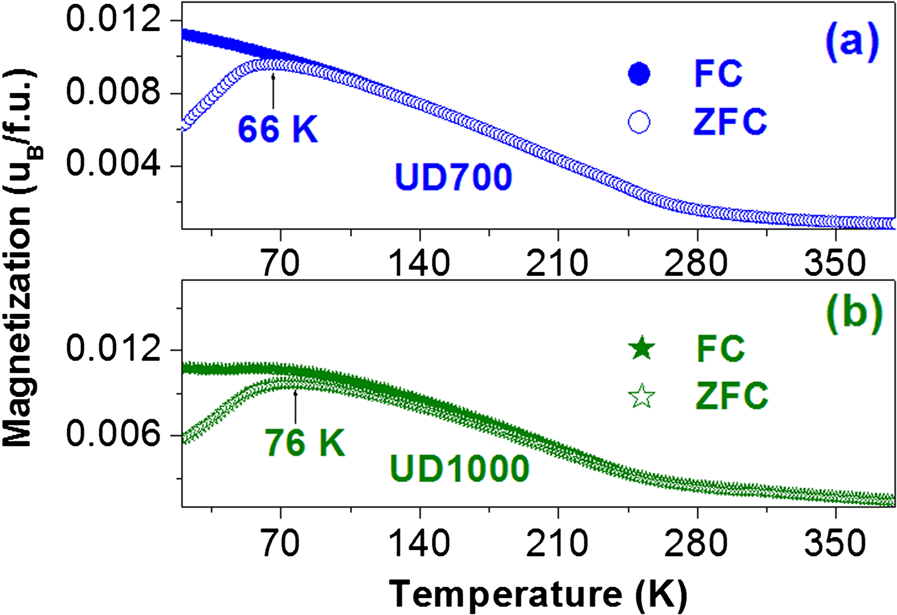
**The magnetization measured in zero field-cooled and field-cooled modes.** The magnetization measured in zero field-cooled (*M*^ZFC^) and field-cooled (*M*^FC^) modes from 20 to 380 K in a field of 200 Oe for the samples UD700 **(a)** and UD1000 **(b)**.

**Figure 5:**
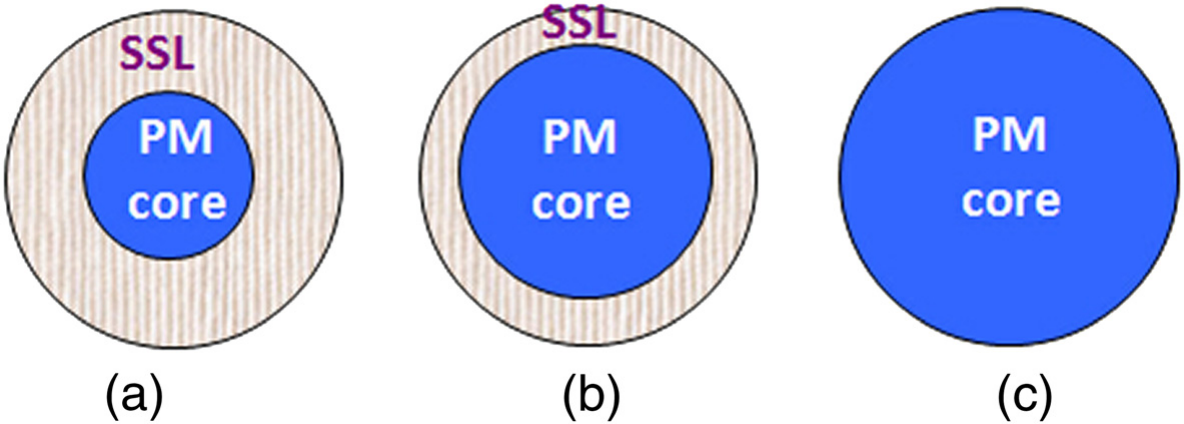
**The schematic plot for understanding the magnetic behavior at 300 K for undiluted samples.** The core-shell magnetization models in the situations annealing at low temperature such as 700°C **(a)**, at intermediate temperature such as 800°C or 900°C **(b)**, and at highest temperature such as 1000°C **(c)**.

Figure 4 shows the magnetization measured in zero field-cooled (*M*^ZFC^) and field-cooled (*M*^FC^) modes from 20 to 380 K in a field of 200 Oe for the samples UD700 (a) and UD1000 (b). For the sample UD700, a peak at 66 K in the *M*^ZFC^ curve is found, usually signifying the blocking temperature (*T*_*B*_) [5,30]. *T*_*B*_ is associated with the particle size (*V*) and the effective anisotropy (*K*_eff_) through *K*_eff_*V* = 25*k*_*B*_*T*_*B*_, where *k*_*B*_ is the Boltzmann constant. At the temperature above *T*_*B*_, the magnetic anisotropy energy barrier is overcome by the thermal energy, and the magnetic moment of each NP randomly fluctuates from one easy direction to another. Thus, the coercive field becomes small, and the small *H*_*c*_ value may result from the surface anisotropy induced by the SSL [31]. This phenomenon is known as SPM. For the sample UD1000, *T*_*B*_ locates at 76 K. The variation of *T*_*B*_ can be assigned to the synergistic effects of the particle volume *V* and the effective anisotropy constant *K*_eff_. According to the XRD results, the crystallite size of UD1000 is 38.8 nm, larger than that (10.7 nm) of UD700, which may result in the increase of *T*_*B*_. According to the above discussion, the magnetic behavior at room temperature can be understood by a core-shell magnetization model, as schematically plotted in Figure 5.

As discussed above, the total moment (*M*_total_) of a particle can be expressed as *M*_total_ = *M*_core_ + *M*_SSL_. At 300 K, the ZLFO core is paramagnetic (PM) (with theoretical molecular moment of 0.1 *μ*_*B*_*/f.u*.). The sample annealed at low temperature (such as 700°C), as shown in Figure 5a, has the small core and the thick SSL. For the sample annealed at higher temperatures such as at 800°C and 900°C, as shown in Figure 5b, the core becomes larger and simultaneously, the SSL becomes thinner. While the sample is annealed at 1,000°C, the SSL becomes thinner or disappears, as shown in Figure 5c, and the *M*(*H*) loop behaves as PM with a linear loop shape in the field range used. Therefore, the gradual decrease in *M*_max_ for the samples UD700, UD800, UD900, and UD1000 can be assigned to the decrease of *M*_SSL_ [32].

**Figure 6:**
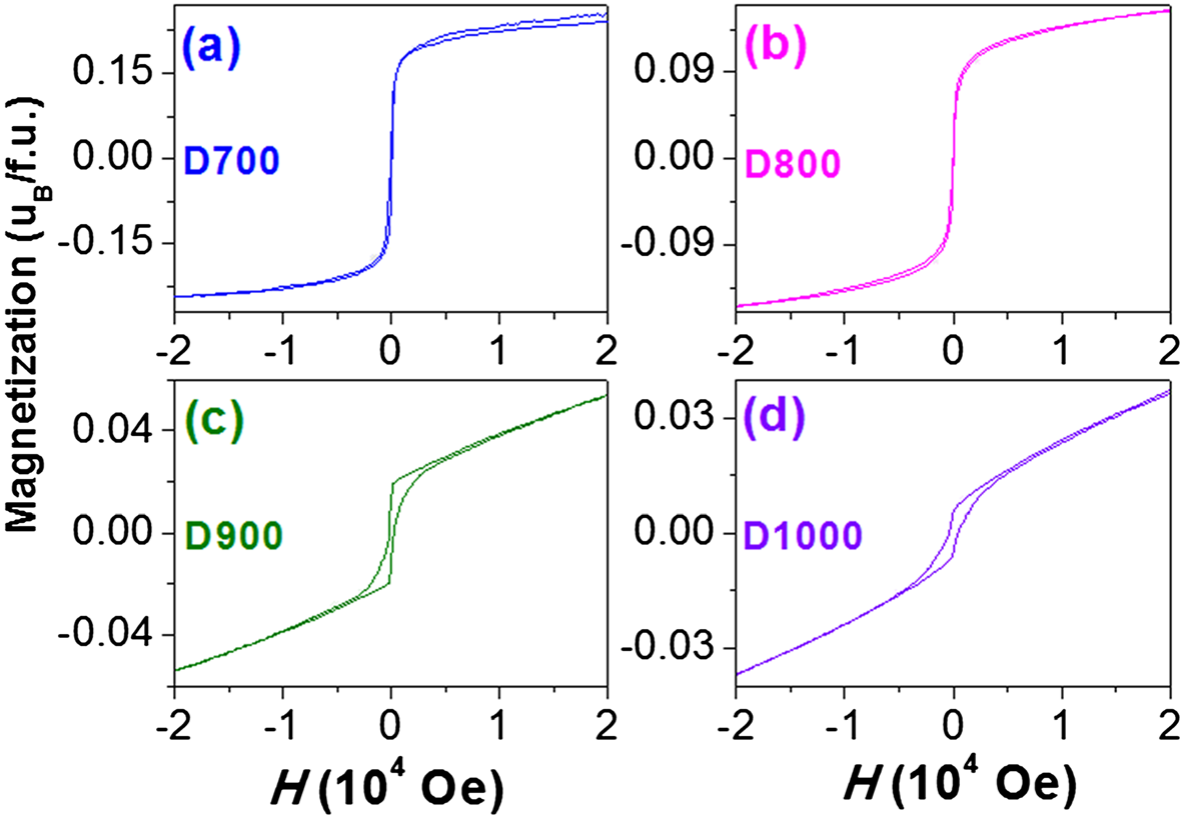
***M*(*H *) loops with the mass-normalized magnetization value. ***M*(*H*) loops of all diluted samples D700 **(a)**, D800 **(b)**, D900 **(c)**, and D1000 **(d)** measured at room temperature.

Figure 6 shows the *M*(*H*) loops of all diluted samples with the magnetization value being normalized according to the mass ratio of ZLFO/Al_2_O_3_. The *H*_*c*_ value is 13, 6, 191, and 391 Oe, and the *M*_max_ value at *H* = 2 T is 0.23, 0.15, 0.05, and 0.04 *μ*_*B*_*/f.u*. The *M*_max_ value also decreases with increasing annealing temperature, which can be assigned to the reduction of SSL thickness, as discussed in Figure 5. For the samples D700 and D800, their *H*_*c*_ values are very small and the magnetization tends to saturate with increasing the *H* value, indicating the characteristic of the SPM. For the samples D900 and D1000, *M*(*H*) loops exhibit the distinct hysteresis and their *H*_*c*_ values are much higher than those of correspondingly undiluted samples, which is characteristic of weak ferromagnetism.

**Figure 7:**
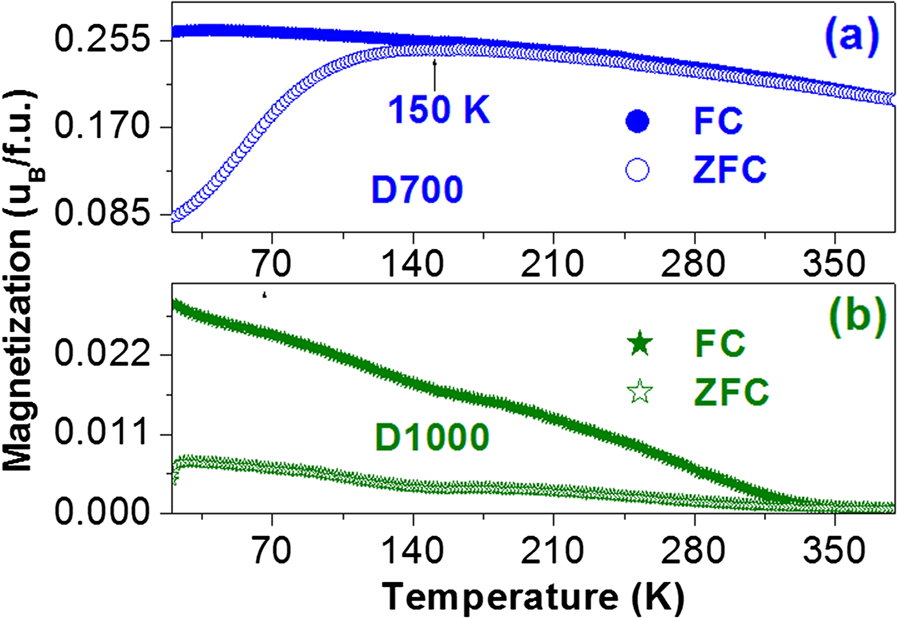
**The mass-normalized magnetization measured in zero field-cooled and field-cooled modes.** The mass-normalized magnetization measured in zero field-cooled (*M*^ZFC^) and field-cooled (*M*^FC^) modes from 20 to 380 K in a field of 200 Oe for the samples D700 **(a)** and D1000 **(b)**.

Figure 7 shows the mass-normalized magnetization measured in zero field-cooled (*M*^ZFC^) and field-cooled (*M*^FC^) modes from 20 to 380 K in a field of 200 Oe for the samples D700 (a) and D1000 (b). A peak in the *M*^ZFC^ curve appears at 150 K for the sample D700, and the *M*^ZFC^ and *M*^FC^ curves almost overlap above 150 K. However, for the sample D1000, the *M*^ZFC^ and *M*^FC^ curves do not overlap until about room temperature, much higher than 150 K, indicating the enhanced irreversibility. After annealed at 1,000°C, the interface between ZLFO and Al_2_O_3_ may form M-O-Al (M = Zn, La, and Fe in ZLFO) bonds. These bonds play a pinning role in the moment reverse, leading to the enhanced irreversibility and consequently resulting in the increase of coercivity, as observed in diluted Co and γ-Fe_2_O_3_ NPs [26,33-36].

## Conclusions

The ZLFO NPs were synthesized by the hydrothermal method. Then, some of ZLFO NPs were diluted in the Al_2_O_3_ matrix through the sol–gel method and the others were undiluted. The undiluted and diluted ZLFO were finally annealed at temperatures of 700°C, 800°C, 900°C, and 1,000°C to investigate the effects of surface spin and interface effects between ZLFO and Al_2_O_3_ on the magnetic parameters and magnetic ordering states.

For the undiluted samples, with increasing the annealing temperature, the thickness of the SSL decreases and ZLFO experiences SPM and PM according to the results of hysteresis loops. The maximum magnetization, *M*_max_, at 2 T of ZLFO decreases with increasing the annealing temperature which can be assigned to the decrease of SSL. For the diluted samples, the surface spin and the interface effect between ZLFO NPs and the Al_2_O_3_ matrix are the dominant factors affecting the magnetic properties. Our results indicate that in the nano-sized magnets with weak magnetism, the surface spin plays a crucial rule in the magnetic properties.

## Competing interests

The authors declare that they have no competing interests.

## Authors’ contributions

STX synthesized ZLFO NPs, performed the measurements, analyzed the magnetic properties, and wrote the manuscript. YQM gave the instruction to this research work, analyzed the results, and wrote the manuscript. YFX, XS, and BQG measured and analyzed the magnetic properties of nanoparticles. GHZ and ZXD supervised the overall study. All authors contributed to discussing the results and writing manuscript. All authors read and approved the final manuscript.
